# Cinnabar Induces Renal Inflammation and Fibrogenesis in Rats

**DOI:** 10.1155/2015/280958

**Published:** 2015-02-05

**Authors:** Ying Wang, Dapeng Wang, Jie Wu, Bohan Wang, Liangjun Wang, Xin Gao, Hai Huang, Honglin Ma

**Affiliations:** Department of Occupational and Environmental Health, School of Public Health, Liaoning Medical University, Jinzhou, Liaoning 121001, China

## Abstract

The purpose of this study was to investigate whether cinnabar causes renal inflammation and fibrosis in rats. Rats were dosed orally with cinnabar (1 g/kg/day) for 8 weeks or 12 weeks. The control rats were treated with solvent (5% carboxymethylcellulose solution) over the same time periods, respectively. Renal mercury (RHg), urinary mercury (UHg), serum creatinine (SCr), urine kidney injury molecule 1 (KIM-1), renal pathology, and renal mediators were examined. At both 8 weeks and 12 weeks, RHg, UHg, and urine KIM-1 were significantly higher in the cinnabar group than in the control group, although SCr was unchanged. Kidney lesions in the cinnabar-treated rats occurred mainly in the tubules and interstitium, including vacuolization, protein casts, infiltration of inflammatory cells, and slight increase in interstitial collagen. In addition, mild mesangial proliferation was observed in glomeruli. Moreover, the expression of inflammatory and fibrogenic mediators was upregulated in the cinnabar group. In conclusion, cinnabar may cause kidney damage due to the accumulation of mercury, and renal inflammation and slight fibrogenesis may occur in rats. In the clinic, the potential risk of renal injury due to the prolonged consumption of cinnabar should be considered even though the agent is relatively nontoxic.

## 1. Introduction

The safety of traditional medicine has become a major public focus [1, 2]. Cinnabar, a naturally occurring red mercuric sulfide (HgS ≥ 96%) [3], has been widely used in Chinese and Indian medicine for the treatment of palpitation, insomnia, nervous disorders, epilepsy, and high fever [4–8]. According to the Chinese Pharmacopoeia (2010 edition), approximately 5% of patented Chinese medicines contain cinnabar, including the classic preparations Angong Niuhuang Wan and Niuhuang Qinggong Wan [3]. Cinnabar is toxic because it contains mercury [9, 10]. However, the toxicity profile is mild because HgS has very low solubility in water, and the occasional cases of cinnabar intoxication mainly result from inappropriate usage [11–15]. The kidney is the primary organ of mercury accumulation after exposure to cinnabar [16–18], and overdose or prolonged use of cinnabar can result in renal dysfunction [11].

Mercury is a well-known nephrotoxic metal, and all forms of mercury (elemental, inorganic, and organic) cause kidney damage [19]. The proximal tubules are preferentially affected by mercury in the kidney [19]. Exposure to mercury can lead to tubular necrosis [20–22], tubulointerstitial nephritis [23, 24], and immune-mediated glomerulonephritis [25–27]. Prolonged exposure may also induce interstitial fibrosis and glomerulosclerosis [28, 29]. Renal fibrosis is the result of a failed wound healing process that occurs after an initial insult [30]. In general, inflammatory reactions occur in the earliest stage of fibrosis, and persistent inflammation is associated with the progression of fibrogenesis [31, 32]. That is, inflammation must be present for fibrosis to occur [33].

A few studies show that renal interstitial inflammatory infiltration and tubular necrosis occur in cinnabar-treated rats [34, 35]. However, whether the renal inflammation caused by cinnabar evolves into fibrosis is unclear. To address this question, we administered cinnabar to rats by intragastric administration and analyzed the Hg content in the kidney and urine and examined renal function and pathology. Furthermore, we measured renal expression of mediators involved in inflammation and fibrogenesis. The results suggested that inflammation and mild fibrogenesis may occur in the kidneys of the cinnabar-treated rats. The potential risk from the consumption of cinnabar should be considered in the clinic.

## 2. Materials and Methods

### 2.1. Animals

Sprague-Dawley rats (160 ± 20 g) were purchased from Vital River Laboratory Animal Technology Co., Ltd. (Beijing, China). All animal experiments were approved by the Ethical Committee of Liaoning Medical University (number Gb05130156). Rats were housed in a temperature-controlled (22 ± 1°C) room, maintained on a 12-hour light/dark cycle, and held in quarantine for 1 week before the experiment. With the exception of overnight fasts before scheduled necropsies, rats were allowed to have free access to food and water throughout the study.

### 2.2. Rat Cinnabar Subchronic Poisoning Model

Rats were randomly divided into the control group or the cinnabar group (eight males and eight females per group). Rats in the cinnabar group were treated via intragastric administration with 1 g/kg/day cinnabar (96% HgS, De-Chang-Xiang Drug Company, Guiyang, Guizhou, China) in 0.5% carboxymethylcellulose solution, which corresponds to 20 times the clinical allowable limit of cinnabar set by the Chinese Pharmacopoeia 2010 edition. Control rats received an equal volume (10 mL/kg/day) of solvent. The cinnabar dose and the duration of treatment were based on previous studies [34, 36]. Clinical symptoms including skin, fur, excretions, behavior, activity, and mortality as well as other clinical signs of toxicity were observed once per day throughout the experiment period, and body weight was measured weekly. Four males and four females in each group underwent necropsy after consecutive dosing for 8 weeks and the remainder underwent necropsy after 12 weeks. Blood and urine samples were collected for examination of renal function and Hg content. Kidney tissues were collected for estimation of mercury residue, histopathology, and mediators.

### 2.3. Determination of Mercury Levels in the Kidney

The mercury standard solution (10^6^ *μ*g/L, National Analysis Center for Iron and Steel, Beijing, China) was diluted to 10^4^ *μ*g/L with 0.5 g/L potassium dichromate. The resulting solution was then diluted with 5% nitric acid to 100 *μ*g/L and further diluted with ultrapure water to generate a series of standard solutions (0 *μ*g/L, 0.5 *μ*g/L, 1 *μ*g/L, 5 *μ*g/L, 10 *μ*g/L, and 15 *μ*g/L).

Renal mercury (RHg) was measured by hydride generation atomic fluorescence spectrometry. Renal samples (0.3 g) were placed into a digestion tube and digested with a solution containing 6 mL nitric acid and 2 mL hydrogen peroxide. The samples were predigested overnight at room temperature, followed by digestion in a microwave oven (CEM, Matthews, North Carolina, USA) as follows: samples were heated to 120°C within 5 min and maintained for 1 min, heated to 160°C within 5 min and maintained for 5 min, and heated to 170°C within 3 min and maintained for 10 min. Maximum power was 1600 W. The fully digested samples were heated to 90°C for 90 min to remove the acids and diluted with ultrapure water to a final volume of 50 mL. Hg content was measured using a hydride generator (MHS15, PerkinElmer, Massachusetts, USA) and an atomic fluorescence photometer (AFS-230E, Kechuang Haiguang, Beijing, China) under the following conditions: lamp current, 10 mA; photomultiplier tube electric voltage limit, –250 V; atomizer height, 10 mm; carrier gas flow rate, 500 mL/min; shielding device flow rate, 1.0 L/min. A standard curve was prepared using the mercury standard series described above. Values were obtained by reading the peak area with a 10 s read time and a 1 s delay time. The blank discriminant value was 2. Each sample was read three times. Standard addition and recovery experiments were conducted to control for the quality of this analysis method.

### 2.4. Determination of Mercury Levels in Urine

The preparation of mercury standard solution was the same as described in Section 2.3. Urinary mercury (UHg) was measured by hydride generation atomic absorption spectrometry. A 2 mL urine sample was placed into a colorimetric tube with a plug and ultrapure water was added to yield a total volume of 10 mL. The sample was blended, and 2 mL potassium permanganate (50 g/L) and 1 mL concentrated sulfuric acid were mixed into the solution and incubated for 5 min at room temperature. The mixture was heated at 50°C for 2 h in a water bath. Hydroxylamine hydrochloride solution (200 g/L) was added dropwise with agitation until the mixture just faded, and the resulting solution was incubated for 30 min (without the plug). Next, 5 mL hydrochloric acid (diluted with an equal volume of ultrapure water) was added to the mixture. Finally, ultrapure water was added to achieve a final volume of 50 mL, and the mixture was blended. Hg content was measured using a hydride generator (MHS15, PerkinElmer, Massachusetts, USA) and an atomic absorption spectrometer (AA800, PerkinElmer, Massachusetts, USA) under the following conditions: sample, 10 mL; reducing agent, 2 mL 3% NaBH_4_; carrier gas, argon; wavelength, 253.7 nm; lamp current, 6 mA; slit width, 0.7 nm. A standard curve was prepared using the mercury standard series described in Section 2.3. Values were obtained by reading the peak height for 10 s. Each sample was read three times. Standard addition and recovery experiments were conducted to control for the quality of this analysis method.

### 2.5. Renal Function Examination

Before the rats were sacrificed, urine was collected for 24 h with a metabolic cage. Rats were anaesthetized by 4% pentobarbital sodium (i.p.), and blood samples (approximately 1.5 mL) were collected from the abdominal aorta into tubes with no anticoagulant for clinical chemistry studies. After sitting for 30 min, blood samples were centrifuged at 3000 rpm for 10 min to obtain serum. Levels of blood serum creatinine (SCr) were detected using an automatic analyzer (7180, Hitachi, Tokyo, Japan).

Urinary concentration of kidney injury molecule 1 (KIM-1) was analyzed with an ELISA kit (Abcam, Cambridge, UK) according to the manufacturer's instructions. Each sample was assayed in duplicate as follows. Prepared standards and diluted samples (100 *μ*L) were added to appropriate wells of the 96-well plate. The plate was incubated at 37°C for 90 min. The contents of each well were discarded, and 100 *μ*L of 1x Biotinylated anti-rat KIM-1 antibody was added to each well and the plate was incubated at 37°C for 60 min. The plate was washed three times, and 100 *μ*L of 1x avidin-biotin-peroxidase complex working solution was added to each well. The plate was incubated at 37°C for 30 min and washed five times. A 90 *μ*L aliquot of prepared TMB color developing agent was added into each well and the plate was incubated in the dark at 37°C for 30 min, after which 100 *μ*L of prepared TMB stop solution was added to each well. The O.D. absorbance was read at 450 nm in a microplate reader (VICTOR3V, PerkinElmer, Singapore) within 30 min of adding the stop solution. The standard curve was plotted as the relative O.D. 450 of each standard solution (*Y*) versus the respective concentration of the standard solution (*X*). The rat KIM-1 concentration of the samples was interpolated from the standard curve.

### 2.6. Renal Pathological Examination

Kidney tissues were fixed in 10% buffered formalin and embedded in paraffin. Sections (5 *μ*m) were cut and stained with hematoxylin and eosin (HE), Masson, and periodic acid-Schiff (PAS). The stained sections were examined under an optical microscope. The degree of interstitial collagen deposition was analyzed according to the Masson-stained sections [37, 38]. Ten tubulointerstitial fields that were randomly selected at ×200 magnification in each rat were assessed according to the percentage of positive staining (blue) area in the field (0, no positive staining; 1, <25%; 2, 25% to 50%; 3, 50% to 75%; 4, 75% to 100%), and the average was taken as the score of interstitial collagen deposition.

### 2.7. Measurement of Renal Inflammatory and Fibrogenic Mediators

Mediators in kidneys from rats sacrificed at 12 weeks were measured using a biotin label-based rat antibody array (RayBio, AAR-BLM-1, RayBiotech, Norcross, GA, USA). First, approximately 10 mg of renal tissue was homogenized in 0.4 mL lysis buffer and centrifuged at 13000 rpm for 10 min. The supernatant was transferred to a dialyzer and dialyzed with 4000 mL PBS buffer (pH 8) at 4°C, stirring gently. The PBS buffer was changed and samples were dialyzed again. Three hours were allowed for each dialysis step. Protein concentrations of the resulting tissue lysates were measured using a BCA kit (Pierce, Rockford, IL, USA). Samples were labeled with biotin using a labeling reagent from the kit. After incubating at room temperature for 30 min, stop solution from the kit was added, and free biotin was removed using a spin column. The column was then centrifuged at 1000 ×g for 3 min to collect the sample. Each membrane was placed into the provided tray and blocking buffer was added. The membrane was incubated at room temperature for 1 h, and the blocking buffer was decanted from each container. The membranes were incubated with samples diluted in blocking buffer at 4°C with gentle shaking overnight. The membranes were then washed with wash buffer from the kit. Following washing, the membranes were incubated with 1 : 8000 diluted IRDye:emoji: 800CW-streptavidin (LI-COR, Lincoln, NE, USA) in the absence of light at room temperature with gentle agitation for 2 h. Finally, bound streptavidin was detected using an Odyssey scanner (LI-COR, Lincoln, NE, USA), and the images were analyzed using the RayBio analysis tool.

### 2.8. Statistical Analysis

Statistical analysis was performed using Student's* t*-tests. Data were expressed as mean ± standard deviation (SD). *P* values less than 0.05 were considered statistically significant.

## 3. Results

### 3.1. General Toxic Effects of Cinnabar

Throughout the experiment, all rats appeared to be in good condition. No abnormality was observed in cinnabar-treated rats except for the presence of red feces, which indicated unabsorbed cinnabar. Kidney to body weight ratio was not significantly different between the cinnabar-treated and control group after 8 or 12 weeks of treatment.

### 3.2. RHg and UHg Were Increased in Cinnabar-Treated Rats

The RHg content directly reflects the mercury accumulation in the kidney, and UHg is an ideal biomarker for long-term mercury exposure. The levels of RHg and UHg are presented in Table 1. RHg was higher in the cinnabar group than in the control group at 8 weeks and 12 weeks (*P* < 0.01, *P* < 0.01, resp.), and UHg was also higher in the cinnabar group than in the control group at 8 weeks and 12 weeks (*P* < 0.01, *P* < 0.05, resp.). In the cinnabar group, UHg was significantly higher in rats treated for 12 weeks than rats treated for 8 weeks (*P* < 0.05), whereas RHg was similar between the 8- and 12-week cinnabar-treated rats (*P* > 0.05).

### 3.3. Cinnabar Damaged Renal Tubular Function in Rats

Urinary KIM-1 is a sensitive marker of proximal tubule injury, and SCr is a classic marker of glomerular dysfunction.  Table 2 shows the changes in urinary KIM-1 and SCr after rats were treated with cinnabar. KIM-1 was increased after 8 weeks and 12 weeks of cinnabar treatment compared to the respective control treatment (*P* < 0.05, *P* < 0.01, resp.). In contrast, SCr did not increase significantly with cinnabar treatment.

### 3.4. Effect of Cinnabar on Renal Histopathology in Rats

**Figure 1:**
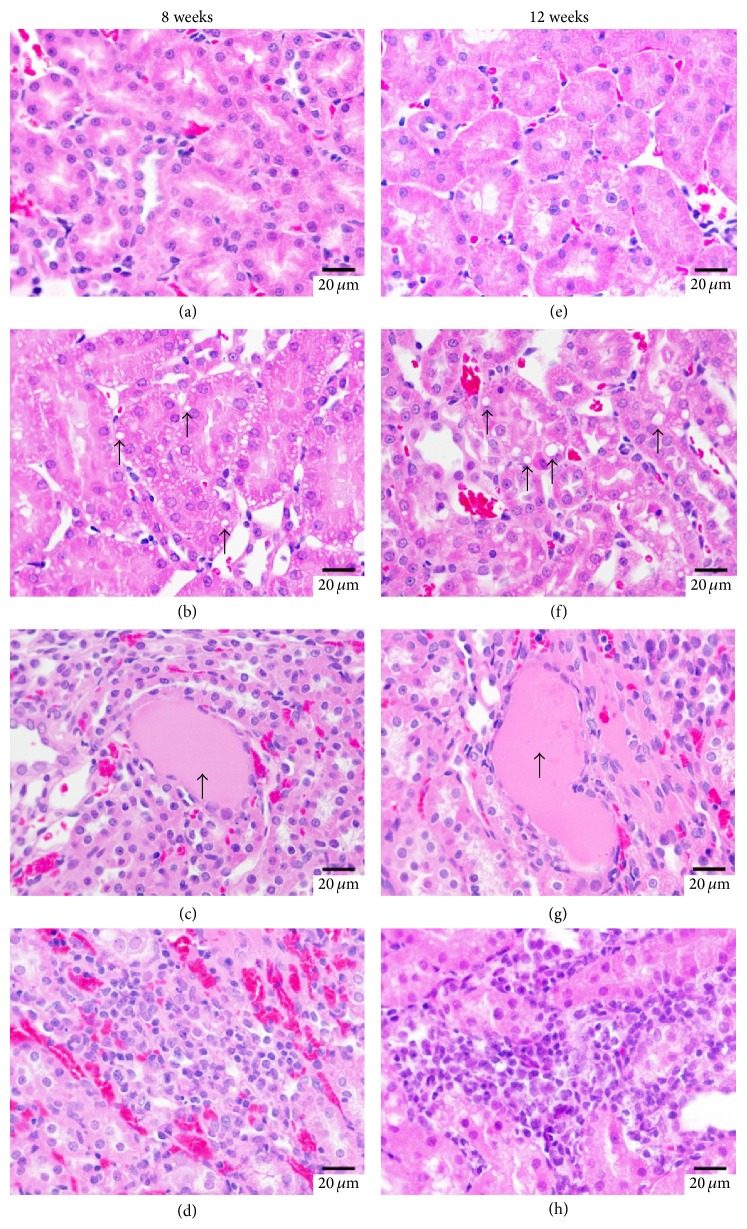
Cinnabar caused tubulointerstitial damage in rats. Renal sections were stained with hematoxylin and eosin (HE). (a) The control group, 8 weeks. ((b)–(d)) The cinnabar group, 8 weeks. (e) The control group, 12 weeks. ((f)–(h)) The cinnabar group, 12 weeks. (a) and (e) show the normal histology of the kidney. (b) and (f) show vacuolization of tubular cells (indicated by arrows), (c) and (g) show protein casts in tubules (indicated by arrows), and (d) and (h) show infiltration of inflammatory cells in cinnabar-treated rats. Scale bar = 20 *μ*m.

**Figure 2:**
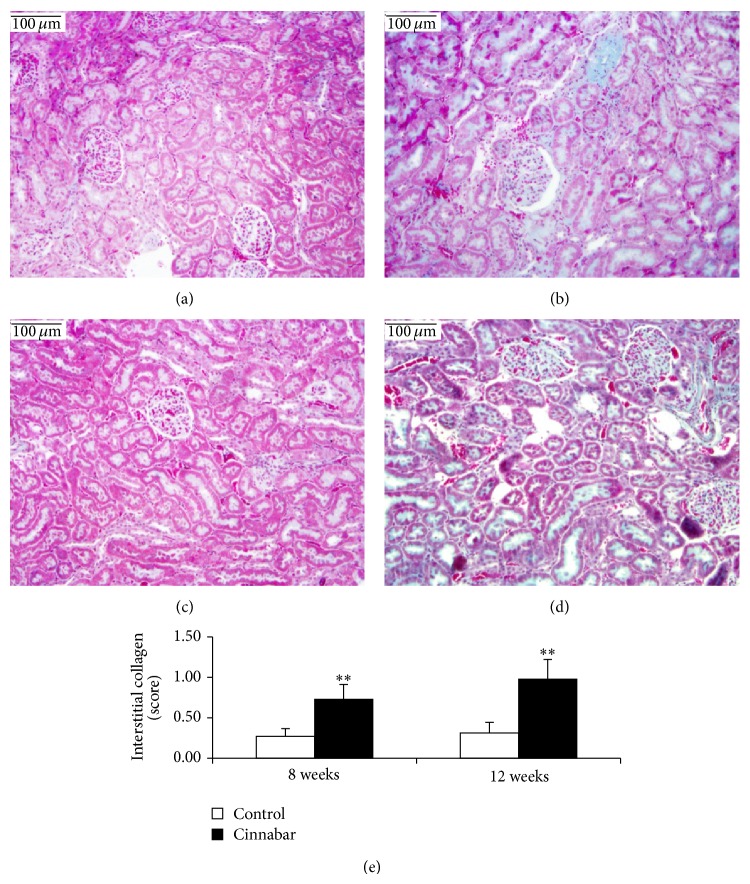
Cinnabar caused mild tubulointerstitial fibrogenesis in rats. Renal sections were stained with Masson. (a) The control group, 8 weeks. (b) The cinnabar group, 8 weeks. (c) The control group, 12 weeks. (d) The cinnabar group, 12 weeks. (e) Bar graph of interstitial collagen (score). Scale bar = 100 *μ*m in ((a)–(d)). ^**^ *P* < 0.01, compared with the control group (*n* = 6).

**Figure 3:**
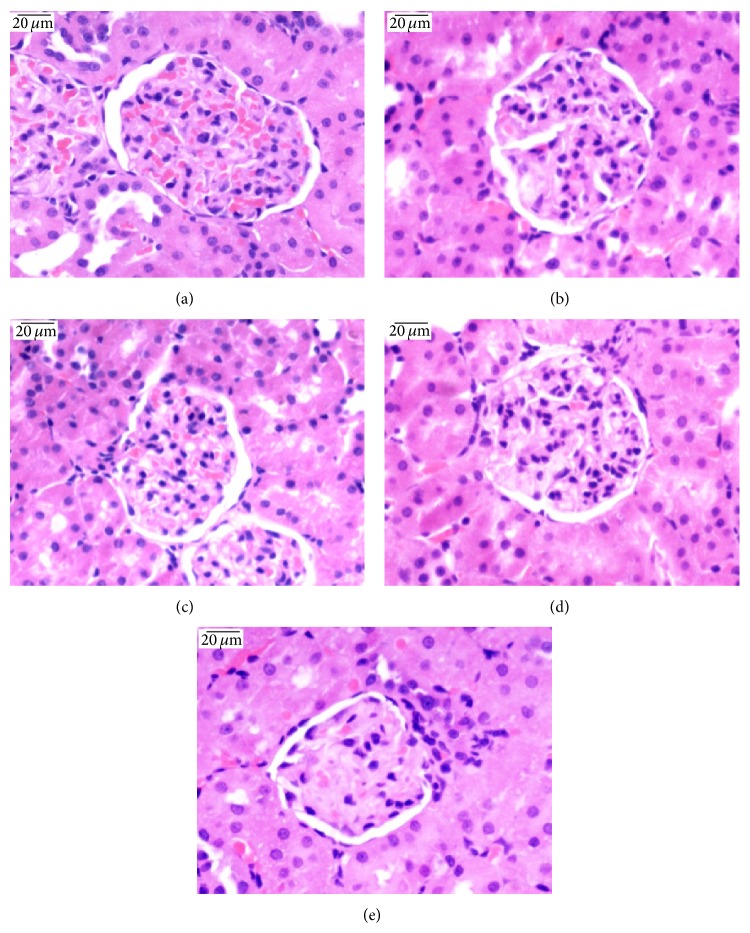
Cinnabar caused glomerular damage in rats. Renal sections were stained with hematoxylin and eosin (HE). (a) Control group, 8 weeks. (b) Cinnabar group, 8 weeks. (c) Control group, 12 weeks. (d) and (e) Cinnabar group, 12 weeks. (b) and (d) show mesangial proliferation. (e) shows focal glomerular sclerosis. Scale bar = 20 *μ*m.

**Figure 4:**
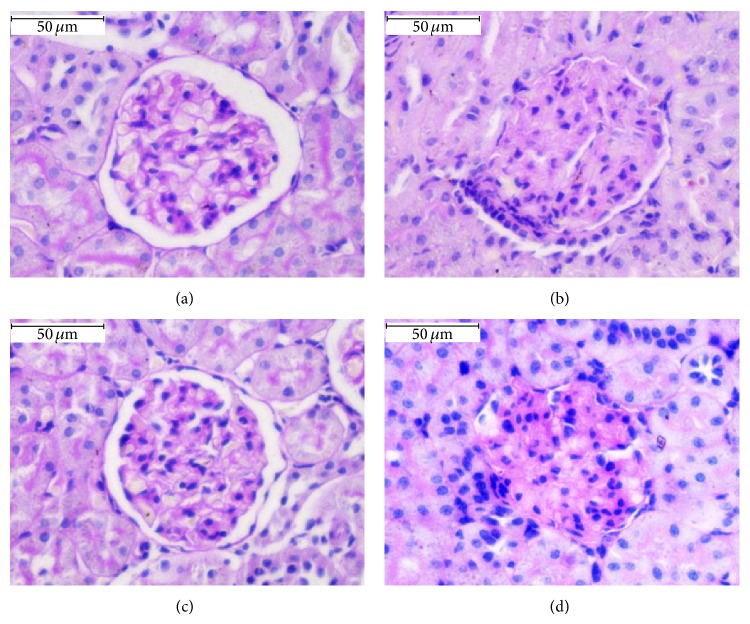
Cinnabar caused mesangial proliferation in rats. Renal sections were stained with periodic acid-Schiff (PAS). (a) Control group, 8 weeks. (b) Cinnabar group, 8 weeks. (c) Control group, 12 weeks. (d) Cinnabar group, 12 weeks. Scale bar = 50 *μ*m.

Kidney sections stained with HE (Figures 1 and 3), Masson (Figure 2), and PAS (Figure 4) were observed under light microscopy. No lesions were found in samples from the control rats. At both 8 weeks and 12 weeks, kidney lesions in the cinnabar-treated rats occurred mainly in the tubules and interstitium, including vacuolization of tubular cells (Figures 1(b) and 1(f)), presence of protein casts in the tubules (Figures 1(c) and 1(g)), infiltration of inflammatory cells (lymphocytes, monocytes, and plasmocytes) (Figures 1(d) and 1(h)), and widened renal tubular gaps (Figure 2(d)). In addition, interstitial collagen was slightly increased in the cinnabar-treated rats compared to the control rats (*P* < 0.01) (Figure 2). Moreover, mild mesangial proliferation (Figures 3(b), 3(d), 4(b), and 4(d)) was seen in glomeruli, and focal glomerular sclerosis (Figure 3(e)) was found occasionally in the cinnabar-treated rats.

### 3.5. Renal Expression of Inflammatory and Fibrogenic Mediators Were Upregulated in the Cinnabar-Treated Rats

To further clarify renal inflammation and fibrogenesis in the cinnabar-treated rats, we measured the expression of mediators in the kidney from the rats treated for 12 weeks using an antibody array. Levels of cytokines associated with inflammation and fibrogenesis were significantly higher in the cinnabar group than in the control group (*P* < 0.05), including B7-1/CD80, GM-CSF, E-selectin, IL-1*β*, IL-6, IL-12/IL-23 p40, IP-10, LIX, L-selectin/CD62L, MCP-1, MDC, MIP-1*α*, MIP-2, MIP-3*α*, FGF-BP, IL-4, IL-5, IL-10, IL-13, MMP-13, PDGF-AA, TGF-*β*1, TIMP-1, TIMP-2, TIMP-3, and VEGF (Table 3).

## 4. Discussion

Cinnabar, an important traditional Chinese medicine, has been used for more than 2000 years. Cinnabar is almost insoluble in water and poorly absorbed in the gastrointestinal tract. The medicine is generally nontoxic at therapeutic doses and is less toxic than HgCl_2_ and MeHg [12–14]. However, cases of cinnabar poisoning have been reported occasionally [9], primarily from overdose, prolonged dosage, and decocting [11, 39]. Such inappropriate usage leads to excessive accumulation of mercury in the body, especially in the kidney. Improper use of cinnabar can lead to kidney damage and even renal dysfunction in serious cases [9]. Inflammation is a defense response to tissue injury, and fibrogenesis is the result of a failed wound healing process that occurs after an initial insult [30]. In this study, we evaluated cinnabar-induced renal injury in rats, focusing on renal inflammation and fibrogenesis.

RHg was increased significantly in cinnabar group than in the control group at both 8 weeks and 12 weeks, confirming that cinnabar was absorbed and Hg was distributed in the kidney, which was the cause of cinnabar-induced renal damage. Although the cumulative intake of cinnabar continued to increase over time, RHg at 12 weeks was similar to that at 8 weeks because UHg was still on the increase. Mercury primarily damages the renal tubules and has also been shown to damage the glomerulus in severe cases [40]. SCr is a classical marker of glomerular function, while urinary KIM-1 is an indicator of renal proximal tube injury [41]. Urinary KIM-1 increased significantly in cinnabar group but SCr did not, indicating that cinnabar preferentially damaged the function of renal tubule.

The main renal pathology changes in the cinnabar-treated rats were the presence of vacuoles in tubular cells, protein casts in tubules, and infiltrated inflammatory cells in tubulointerstitium, which was consistent with the literature [34]. In addition, slight interstitial fibrogenesis and occasional focal glomerular sclerosis were observed in the cinnabar group. To further analyze the progression of fibrogenesis, we measured the expression of mediators related to inflammation and fibrogenesis. The results showed that chemokines, selectins, GM-CSF, ILs, and tissue inhibitors of metalloproteinase (TIMPs) were higher in the cinnabar group than in the control group.

Leukocytes are potently attracted to chemokines. In the cinnabar group, the expressions of MCP-1/CCL2, MIP-1*α*/CCL3, MIP-3*α*/CCL20, MDC/CCL22, MIP-2/CXCL2, LIX/CXCL5, and IP-10/CXCL10 were upregulated. Monocytes are mostly attracted to CCL chemokines, whereas neutrophils tend to target CXCL chemokines [42]. Moreover, chemokines also recruit T cells to kidneys affected by chronic kidney injury [42]. The increased expression of these chemokines may explain the recruitment of inflammatory cell in tubulointerstitium. L-selectin is present on leukocytes while E-selectin is expressed on activated endothelial cells [43]. Binding of L-selectin and E-selectin to their respective ligands mediates the initial capture and rolling of leukocytes on endothelial cells, which is required for the migration of leukocytes to inflammation sites [43]. The increased expression of L-selectin and E-selectin may promote the migration of leukocytes to the injury site in the cinnabar-treated rats. In addition, GM-CSF is upregulated, which increases the phagocytic and microbicidal activity of neutrophils and macrophages and induces their production of proinflammatory cytokines [44].

IL-1*β* and IL-6 were elevated in the cinnabar-treated rats, which contributed to both acute and chronic inflammation [45]. Acute inflammation is a recognized part of normal wound healing [46]. However, sustained or repetitive injury causes these normal wound healing responses to persist or become dysregulated, resulting in pathologic deposition of ECM [47]. Fibrogenesis is a dynamic process in which inflammation, tissue remodeling, and tissue repair processes occur simultaneously [48], and in which Th1/Th2 immune response may play a prominent role. Traditionally, Th1 cells are thought to mediate tissue damage, whereas Th2 cells are linked with fibrogenesis [31]. Th1 and Th2 cytokines play opposing roles in fibrosis, as Th1 cytokines suppress fibrogenesis, and the Th2 cytokines are profibrotic [31]. Both the Th1 cytokine (IL-12) and the Th2 cytokines (IL-4, IL-5, IL-10, and IL-13) were upregulated in the cinnabar group, which indicated that Th1/Th2 immune response involved cinnabar-induced renal injury. In the current study, the rats were treated repetitively with cinnabar for 12 weeks. Renal injury and repair occurred simultaneously in rats during the experiment, which was consistent with the increased Th1 and Th2 cytokines. If rats continued breeding after cessation of dosing, renal damage would be lessened and tissue repair would be dominated. Thus, we supposed that the expression of Th2 cytokine would be further increased and renal fibrogenesis would be aggravated in recovery phase.

Fibrogenesis is the result of imbalance between ECM production and degradation. TGF-*β*1 is a premier profibrogenic cytokine, which primarily suppresses collagen degradation and stimulates matrix-producing cell proliferation and collagen synthesis in the repairing tissue [49, 50]. TIMPs specifically modulate the activity of matrix metalloproteinases (MMPs), which play a major role in ECM degradation [30]. Upregulation of TIMPs can inhibit activity of MMPs and lead to reduced ECM degradation. The increase in TGF-*β*1 and TIMPs may contribute to the mild fibrogenesis in the kidney of the cinnabar-treated rats.

## 5. Conclusions

In summary, exposure to cinnabar may cause kidney injury due to accumulation of mercury and cinnabar preferentially damaged renal tubule function in rats. Renal inflammation and slight fibrogenesis may occur in cinnabar-treated rats. Although adverse effects of cinnabar at therapeutic doses are rare and largely tolerable, the potential renal toxicity should be considered particularly when cinnabar is consumed over a prolonged period.

## Figures and Tables

**Table 1:** RHg and UHg increased in cinnabar-treated rats.

Group	*n*	8 weeks		12 weeks	
		RHg (*μ*g/g)	UHg (*μ*g/L)	RHg (*μ*g/g)	UHg (*μ*g/L)
Control	6	0.352 ± 0.319	5.664 ± 2.356	0.404 ± 0.195	3.279 ± 2.723
Cinnabar	6	2.127 ± 0.566^**^	18.117 ± 5.665^**^	2.222 ± 0.652^**^	94.805 ± 64.871^∗#^

RHg: renal mercury. UHg: urinary mercury. Results represent mean ± SE of six animals per group. ^*^ *P* < 0.05, ^**^ *P* < 0.01, compared with the control group. ^#^ *P* < 0.05, compared with cinnabar group sacrificed at 8 weeks.

**Table 2:** Urinary KIM-1, but not SCr, increased in cinnabar-treated rats.

Group	*n*	8 weeks		12 weeks	
		KIM-1 (ng/g)	SCr (*μ*mol/L)	KIM-1 (ng/L)	SCr (*μ*mol/L)
Control	6	96.97 ± 7.07	26.22 ± 5.72	99.41 ± 6.77	28.45 ± 6.20
Cinnabar	6	114.02 ± 11.56^*^	25.93 ± 6.68	120.62 ± 11.86^**^	30.27 ± 5.66

KIM-1: kidney injury molecule-1. SCr: serum creatinine. Results represent mean ± SE of six animals per group. ^*^ *P* < 0.05, ^**^ *P* < 0.01, compared with the control group.

**Table 3:** Renal expression of inflammatory and fibrogenic mediators were upregulated in cinnabar-treated rats.

Mediators	Control	Cinnabar	*P* value
L-selectin/CD62L	101.03 ± 8.19	191.49 ± 19.38	0.0001
E-selectin	139.94 ± 21.33	192.99 ± 22.28	0.0138
MCP-1/CCL2	217.94 ± 34.00	311.54 ± 27.72	0.0053
MIP-1*α*/CCL3	387.63 ± 56.64	514.61 ± 45.24	0.0128
MIP-3*α*/CCL20	219.68 ± 29.32	301.92 ± 14.01	0.0023
MDC/CCL22	401.28 ± 48.74	538.09 ± 55.00	0.0098
MIP-2/CXCL2	364.42 ± 44.82	485.86 ± 39.52	0.0066
LIX/CXCL5	142.33 ± 15.65	243.99 ± 22.39	0.0003
IP-10/CXCL10	197.75 ± 21.82	264.08 ± 29.11	0.0107
GM-CSF	227.82 ± 28.81	331.11 ± 21.78	0.0012
B7-1/CD80	66.01 ± 4.84	99.40 ± 13.17	0.0031
IL-1*β*	195.83 ± 22.85	267.32 ± 27.98	0.0075
IL-6	185.21 ± 26.40	269.65 ± 18.67	0.0020
IL-12/IL-23 p40	212.44 ± 28.69	303.58 ± 34.97	0.0069
IL-4	117.49 ± 10.71	192.26 ± 21.39	0.0008
IL-5	237.21 ± 36.82	351.26 ± 45.17	0.0079
IL-13	173.70 ± 20.62	213.51 ± 15.71	0.0219
IL-10	203.56 ± 32.10	299.32 ± 25.48	0.0034
TGF-*β*1	316.90 ± 30.10	377.74 ± 26.60	0.0231
PDGF-AA	241.43 ± 39.73	363.32 ± 47.78	0.0078
VEGF	278.59 ± 41.53	385.97 ± 44.29	0.0123
FGF-BP	154.79 ± 9.50	258.50 ± 42.18	0.0030
MMP-13	132.97 ± 11.80	174.35 ± 10.65	0.0020
TIMP-1	127.21 ± 10.83	185.38 ± 14.28	0.0006
TIMP-2	138.76 ± 10.70	257.97 ± 8.85	0.0000
TIMP-3	50.96 ± 10.56	91.62 ± 14.49	0.0040

Rats were dosed with cinnabar (1 g/kg/day) for 12 weeks, and levels of cytokines in kidneys were measured using an antibody array. Each level of expression was normalized to that of the positive control. Results are expressed as the mean ± SD (*n* = 4 rats per group). Statistical analysis was performed using Student's *t*-test. Abbreviations: CCL, CC chemokine ligand; CXCL, CXC chemokine ligand; FGF-BP: fibroblast growth factor binding protein; GM-CSF, granulocyte-macrophage colony-stimulating factor; IL, interleukin; IP-10, interferon-inducible protein 10; MCP, monocyte chemotactic protein; MDC, macrophage-derived chemokine; MIP, macrophage inflammatory protein; MMP: matrix metalloproteinase; PDGF-AA: platelet derived growth factor-AA; TGF: transforming growth factor; TIMP: tissue inhibitor of metalloproteinase; VEGF: vascular endothelial growth factor.
